# Bibliographical Notices

**Published:** 1847-01

**Authors:** 


### BIBLIOGRAPHICAL NOTICES.

*Julii Vogel Icones Histologiæ Pathologicæ Tabulæ Histologiam Pathologicam illustrantes viginti sex tabulæ, continentes ccxci figuras, quarum cclxx ad naturam delineatæ sunt.* Lipsiæ, 1843.

*Erlauterungstafeln zur Pathologischen Histologie mit vorzüglicher Rucksicht auf sein Handbuch der pathologischen Anatomie* herausgegeben von Dr. Julius Vogel, ausserordentl. Professor der Medizin in Göttingen. Sechs und zwanzig Tafeln, mit 291 Figuren, wovon 270 nach der Natur gezeichnet sind. 4to. pp. 128. Leipzig, 1843.

*The Pathological Anatomy of the Human Body.* By Julius Vogel, M. D. Professor of Clinical Medicine at the University of Giessen. Translated from the German, with additions By George E. Day, M. A. and L. M. Cantab. Member of the Royal College of Physicians; Physician to the Western General Dispensary; Lecturer on Histology and Animal Chemistry at the Middlesex Hospital Medical School; Member of the Pathological Society of London, and formerly Senior President of the Royal Medical Society of Edinburgh. Illustrated by upwards of one hundred plain and coloured Engravings. 8vo. pp. 587. London, 1847.

In the last volume of the Examiner, (p. 239,) we heralded the advent of the "Icones" of Vogel—the first work on histological pathology that had been issued. The "Pathological Anatomy of the Human Body" by the same author, of which we have before us the translation by Dr. Day, is illustrated by plates taken from the "Icones;" and is, as the translator remarks—or rather implies—the only work in the language that embraces the recent discoveries effected by chemistry and the microscope. It is a treatise on general pathological anatomy; and, we are told, will very shortly be followed by another, devoted to the consideration of pathological changes affecting special organs.

The work consists of an Introduction treating of the relations of pathological anatomy to the other departments of medical science; and of ten chapters, which consider—I. The abnormal development of gaseous matters—Pneumatoses. II. Abnormal collections of aqueous fluids—Dropsies. III. Pathological relations of the blood. IV. The general relations of pathological epigeneses, [new formations.] V. Special relations of pathological epigeneses. VI. Pathological changes in the physical properties of the tissues and organs of the body. VII. Combinations of morbid elementary changes. VIII. Independent organisms in the human body—Parasites. IX. Congenital modifications of the human body—Malformations. And X. Changes occurring in the body after death—Post-mortem changes.

It is obviously impossible for us, in the space we can assign to this notice, to examine into the views of the author on all these important subjects; but we may refer to a few. Under Pneumatoses he properly remarks, that gas may be developed in the human body partly from food in the act of decomposition in the intestinal canal, and partly from the decomposition of the constituents of the body itself. He has no doubt, too,—nor have we,—that it may be actually secreted by different parts of the frame.

"Thus, Magendie and Girardin assert, that on confining a portion of the intestine of a live dog between two ligatures, in the course of some hours the included portion was found full of air, which escaped with a hissing sound on making an incision.* In the intestinal canal of swine we sometimes meet with considerable accumulations of gas between the layers forming the walls of the bowels. Sir Francis Smith† has described an interesting case of the development of gas in man, which deserves a full notice. He states—"On the 12th of May, 1840, I was consulted by a gentleman, who told me that he often suffered from an enormous development of gas in the stomach, which he discharged by eructation: that he likewise, occasionally, experienced a development of gas from the bladder, and that his skin acted in a similar manner, as he had observed in the bath. On the morning of the 15th, I found my patient in a bath at 79° F.

* Magendie et Girardin; Recherches physiolog. sur les gaz intestin. Paris, 1824. p. 24; Lobstein, Path. Anat. Vol. 1, p. 138.

† Dublin Med. Journal, January, 1841. p. 454.

His breast, shoulders, abdomen, and hands, were literally covered with minute bubbles of gas. On being questioned, the attendant at the bath stated that he had never previously witnessed anything of the kind. On removing the hands and arms from the water, the air bubbles disappeared, but gradually returned on again immersing those organs. The bubbles were of the size of a pin's head. On wiping them off, they disappeared, but gradually formed again.

"In opposition to the above observations of Magendie and Girardin, it may be urged that the gas which was developed might probably have arisen from the decomposition of remnants of food in the inclosed portion of intestine, or that the portion of gut becoming distended by peristaltic motion had imbibed air from the peritoneal cavity, or from the adjacent portions of the intestinal canal; and similarly the escape of air from the stomach and urinary bladder, in Smith's case, admits of the same mechanical explanation as has been given in a previous page. Not so, however, the escape of air from the skin: the fact that all bodies, when immersed in water, give off a little entangled air, affords no explanation of the continuous evolution of gas from the skin: neither does the accumulation of air occasionally noticed in the intestinal canal of swine seem to admit either of a mechanical or chemical solution. If we are asked for the particular causes of these developments of gas, I confess I can give no satisfactory reply. No secretion of gases occurs in the human body in a normal condition; for the development of gas in respiration is a purely physico-chemical proceeding, and is in exact accordance with the laws of displacement and diffusion of gases, as has been recently proved by Valentin and Brunner;* and, probably, the same law holds good for the development of gas through the skin. We can only refer to the analogical proceeding in fishes, where we find an actual secretion of gas in the swimming-bladder, and must, for the present, defer all further questions respecting their causes or pathological indications." p. 32.

The most important deviations in the condition of the blood, are described to be as follows:

"1. Its physical and chemical characters may undergo alteration. It may be either thinner or thicker than usual, and its colour may be affected. The blood-corpuscles may appear changed. The proportion of its constituents to each other may be altered, and it may contain matter not normally existing in it, as sugar, free lactic acid, &c.

* Valentin, Lehrb. d. Physiol. d. Menschen, vol. 1, p. 559.

"2. Its quantity may be increased (hyperæmia or polyhæmia) or diminished (anæmia or hypæmia.) This increase or diminution may either be general, extending to the whole organism, or local, and restricted to particular parts of the body.

"3. It may be effused in consequence of laceration of the blood-vessels, into the interstices of the parenchyma of certain organs, or into some of the cavities of the body, constituting extravasation.

"4. The hæmatin may, by a process of decomposition, become dissolved, and then be imbibed by the tissues." p. 59.

The blood also becomes changed by the absorption of *ferments* and other extraneous matters, by which its character is at times essentially altered; and this, after all, is one of the most important considerations to be borne in mind in regard to the blood as a source of disease, and as suggesting methods of medication in various pathological states now properly esteemed of blood-origin. The pathological relations of the blood are, however, not easy of appreciation; but, for that very reason, they should be more closely investigated.

From the chapter on Independent organisms in the human body—Parasites—we extract the following in relation to *Epiphytes* or parasites derived from the vegetable kingdom.

"All the parasitic plants which, up to the present time, have been observed in the human organism, belong to the lowest forms of vegetation—the algæ and the fungi. They are all very minute, so that, to the unaided eye, the greater number are totally invisible, and others are only perceptible when accumulated in large masses. In order to recognize their peculiar structure, and thus to arrive at a more accurate diagnosis, the microscope is invariably necessary, and very high powers are often required. They are found either upon exposed surfaces, namely, upon the skin and mucous membranes, or floating in the fluids of the body. I am acquainted with no authentic case in which they have been observed during life in the parenchyma of human organs. Respecting the origin of vegetable parasites, there are the same two different views which have been noticed in relation to the origin of parasites generally. Whilst, for instance, Kützing,* who has devoted much of his attention to the lower algæ, maintains, that their origin by repeated spontaneous generation is possible, others limit their origin to the mode by

* Phycologia generalis, Leipz. 1843, p. 129, &c.; or his remarks in Erdmann's Journal f. prakt. Chemie, 1837, vol. xii. p. 391.

propagation. Although a positive decision of this disputed question may at present be impossible, it nevertheless appears to me that there are overwhelming reasons in support of the view that they invariably owe their origin to propagation alone. These reasons are chiefly founded upon the researches of Schwann on fermentation, upon similar investigations of Holmholtz,* and upon others which Dr. Merklein has abundantly instituted upon this subject, all which show, that under conditions which otherwise prove favourable to the formation of fungi and algæ, these do not present themselves, when the possibility of the transference of uninjured germs is precluded. Moreover, all the parasites hitherto observed increase in enormous ratios by means of germules or spores: the latter are so infinitely numerous, so minute, and maintain their germinating power so tenaciously against the most common external agents, that by means of water and currents of air they certainly become universally diffused, and can, therefore, develop themselves wherever they meet with favourable conditions. That we have hitherto, in most instances, failed to demonstrate the origin of fungi by transference of germs, can be no argument against this mode of propagation; for, even in the most careful examination, certain fungus-spores, whose diameters are sometimes less than the 1000th of a line, may, and indeed always will escape the notice even of the most practised observer. In some cases the transference of parasitic fungi, or of their spores, from one subject to another, becomes facilitated by distinct relations, as immediate contact, &c., as may occur in porrigo, in some forms of impetigo, mentagra, &c. These are the cases which are especially regarded as contagious. In general, however, peculiar conditions appear requisite for the development and increase of the transferred germs—conditions which are in general only realized by pathological relations. It appears, for instance, that the surface upon which they are to develop themselves must in general, if not always, be in a certain state of chemical decomposition (putrefaction or fermentation;) as, indeed, we find that externally to the human and animal organisms, most fungi are developed only on putrefying substances. Experience shows us, that parasitic fungi are especially liable to occur on foul ulcers, and probably only exist on the skin or mucous membrane in the cases where these are furnished with a layer of decomposing exudation. Parasitic plants have so far a diagnostic value, that they indicate that a process of decomposition is going on, however locally circumscribed it may be. Hence it follows that they do not become developed at all spots on which the germs are deposited: their growth indicates a certain morbid disposition. This view

* Müller's Archiv. 1843, p. 453, &c.

is opposed by the results of experiments made with the view of showing that parasitic plants can be transferred by inoculation to apparently healthy organisms, and then give rise to morbid phenomena: thus, for instance, Hassal* was able to transfer, by inoculation, parasitic fungi from diseased to healthy lettuces, in which they produced the same disease, (softening of the stem.) These cases, however, prove but little: they merely show, that in some instances the disposition to fungoid development need not be great; and they are, moreover, open to the objection that the plants which were inoculated having, perhaps, the same *habitat*, and living under similar relations, already bore within them the morbid disposition." p. 428.

Dr. Vogel then describes the different fungi found in animal fluids—the yeast plant, and sarcina ventriculi; and next the parasitic fungi found on the integument, and its appendages, of man—those of porrigo, mentagra, plica polonica, and of the mucous membranes; after which he treats of the different parasitic animals; terminating with a learned view of malformations, in which he includes the different forms of monstrosity.

The plates, ten in number, are well executed, and the whole work is exceedingly instructive. It indicates on every page that the author is a well read, painstaking, and accurate observer; fully aware of what has been done in other countries as well as his own; and always anxious to do justice to his brethren who have been engaged in the same labours. We shall look with anxiety for the promised volume on special pathological anatomy, which we doubt not will be worthy to take its place alongside the one before us.

Dr. Day—the translator—appears to have executed the duty—if not elegantly—faithfully. He modestly states in the preface, that the additions he has made are trivial and unimportant, with the exception of the plates and their explanations. "These," he remarks, "are almost entirely selected from the author's 'Icones Histologiæ Pathologicæ,' and will, I trust, be found valuable aids to the clear understanding of the subjects they are intended to illustrate." p. xx. We wish he had added "Tabula xii" of the "Icones"—"*Epizoa atque Entozoa Hominis*," in which the different parasites are delineated. The other plates,

* Froriep's N. Notizen. Oct. 1843, p. 54, &c.

referring—as they do—to lesions of special organs, will doubtless appear in the next volume.

We are glad to find, that this valuable treatise is about to be reprinted in this country by the enterprising house of Lea & Blanchard of this city.

---

*Medical Instruction in the United States: an Address delivered to the students of the Philadelphia Association for Medical Instruction, at the close of the Session of* 1846. By Alfred Stillé, M. D., Lecturer on Pathology, and the Practice of Medicine.

In this address, Dr Stillé controverts the assertion of Professor Paine "that American physicians *greatly surpass* all other nations, not only in the *decision*, but in the *success* of their practice," and argues that in attainments, so far from being equal to those of the different nations of Europe, they are greatly behind, and therefore cannot possibly *surpass* them in *decision* or *success* in the treatment of disease.

After contrasting the qualifications for entering upon the study of medicine, demanded in various sections of Europe, with the looseness which prevails in the United States, the lecturer makes the following statement:

"After all the preparatory study we have described, what term of attendance on medical lectures is required of European students? In Austria and France *fifty* months; in Prussia and the secondary states of Germany *forty* months; in Great Britain and Ireland *twenty-four* months; while in the United States, we undertake to produce a competent physician in *eight* months, or one-third of the time deemed necessary by the lowest of the European schools."

In this statement the system of medical education pursued in the United States, it strikes us, is not quite fairly compared with what obtains in Europe. With us, *three years* study we believe is demanded by all the colleges, including attendance on lectures. The student in his attendance on lectures, instead of being confined to two or three subjects in different years, thus extending the term to four or five years, is occupied with all the branches at once, so that in two courses of four months each he may hear as many lectures on certain subjects as would consume double the period of time when but half the number are given. Whether the number of subjects taught simultaneously is more than the mind of an industrious student can master is another question. Every one, who attends but two lectures a day, will not learn more of these particular subjects than others who are engaged with six; and therefore, according to our republican notions of justice, the industrious man, who can acquire a competent knowledge of his profession to commence the practice of it in *three* years, should not be subjected to the expense of a protracted pupilage, because the drone and the dolt may require *five* or *seven.* With us, it must be recollected, no part of a medical student's expenses are borne by government, as in continental Europe, and in the yet infant state of our country the means of parents generally are not adequate to great expense in the education of their sons; nor does government, in most of the states, afford to the regular physician any protection against competition from the uneducated.

There is a wide difference in these respects, between our situation and that of the old institutions and despotic governments, to which Dr. Stillé refers as patterns for our imitation.

Under the circumstances in which we are placed, it would be both unjust and impolitic to prescribe a longer time or greater outlay than experience may show to be indispensably necessary for the accomplishment of the desired object. What may be the shortest time necessary is a question about which men of equal experience will differ; but as the object of all hese requirements is to insure a competent amount of knowledge for the safe and judicious exercise of the office of a physician, and as this can be arrived at by a proper examination, *and in no other way*, there ought to be no difference of opinion on this point. If every aspirant for admission into the ranks of the profession were subjected to this test, there would be little occasion for any other. There need be no inquiry *where* he got his knowledge, nor how *long* he was about it. But here lies the difficulty. The completeness of the examination must depend upon the character of those who are to conduct it. Who shall these be? In order to arrive at a correct appreciation of this matter, we must first look to the form and operation of our government, for no voluntary or self-constituted board, whether examiners or those by whom they are appointed, can exercise any effective control or jurisdiction in the case. Of this fact, there can be no doubt. The federal government possesses no power under the constitution to regulate the business pursuits of the citizens of the States. Every thing of that kind is left with the States respectively. This is the practice as well as the theory of our government. Every state in the union, then, possesses exclusive power over this subject within its limits. But most of the states have refused, absolutely, to restrict the practice of medicine, or to prescribe any qualifications whatever for those practising it, leaving to the common law the task of guarding their citizens by suits for malpractice.

The states respectively have the power, and many of them have exercised it, of establishing colleges for giving instruction and granting degrees. Will these colleges surrender the business of teaching to other and irresponsible associations? That would be to neglect and refuse to perform the duties of a public trust, for which the law has instituted them; and if the existing faculties were to take that course, there is little doubt but that the trustees who govern these institutions would soon find other occupants for their chairs, even among those most eager for reform.

Will the faculties and trustees of the colleges decline to grant diplomas or testimonials of competency to their pupils, whose deportment and acquirements have justly earned them? That is not likely. It would be surrendering the common privilege possessed by all men, of rewarding merit. It would be denying to themselves the right possessed and exercised by every school master and master mechanic, of testifying to the good conduct and proficiency of their pupils and apprentices. Until some legal enactments can be had, prescribing who may practice, and by whom they shall be examined, which is not likely to occur in many of the states in any short period, we apprehend that the present system of examination will continue, and that the character of those who shall be recognised as members of the medical profession will depend on the more or less general diffusion of education and the character of the professors in the different medical schools; whilst the character and fitness of the latter will depend upon whether the honors and emoluments of their stations shall afford adequate inducements to properly qualified individuals to accept the appointments. That such will continue long to be the case may be doubted, when we see the facility with which charters are obtained, and the genera rage for engaging in the business. If the system of medical education is to be improved, and the respectability of the profession maintained, the number of medical colleges must be limited to the actual wants of the community. How this is to be attained it is difficult to say. Most likely the college fever will be allowed to run its course, until a crisis ensues from the complete exhaustion of all means of support. In some instances, like too many plants in one parterre, some will wither and die, whilst others, deriving additional strength and support from their decay, will bring forth better and more abundant fruit.

Notwithstanding the unfavourable picture of us painted by Dr. Stillé, we very much doubt whether we are really so much worse off, or the physicians of the United States are in practical tact and usefulness so much in arrear of their European brethren. Take, for example, the following sketch of matters in England, quoted by him from a work by Mr. Surgeon Wilde, of Dublin: "In England, with few exceptions, (and even in those exceptions the kind of instruction is very meager) there is little or no preparatory education required by the different colleges and licensing bodies. The student is at perfect liberty to choose what lectures, or how many, he will first attend; the object being not how he can best prepare his mind by initiatory degrees, for the more mature branches of study, but how he can soonest, easiest, and cheapest, become possessed of the *certificates of attendance* on the lectures he has never heard. There are no tests required as to his knowledge of any of the subjects he is *supposed* to study till the hour of his examination—and when this examination does arrive, the chances that he is never asked a question, except upon anatomy and surgery, and a little physiology, are, in the chief licensing institutions of Great Britain, so slight as almost to amount to a certainty."

If there is any institution for granting degrees in the United States where the business is conducted as badly as this picture represents, we are wholly ignorant of it.

But the author of the lecture says they manage things better in France, in Prussia, and in Austria. The two last of these governments are units,—absolute monarchies; and can therefore enact what edicts they please, and enforce them throughout their entire kingdoms; which makes a wide difference between their condition and ours. But are the medical men of these countries so much superior to those of Great Britain? Who among all their physicians has done more for our science than Sydenham, Harvey and Jenner; of their surgeons, who more than Pott, Hunter, Cooper, and Brodie; among their chemists, who more than Cavendish, Davy and Dalton? We are aware that a few bright examples are not fair representatives of the whole; but how is it with the mass? Are the practitioners of Great Britain inferior in skill and capacity, as a general rule, to those of France and Germany? We think Dr. Stillé, with all his predilections on this subject, will not assert it. Great learning and the capacity for great usefulness do not mean one and the same thing, nor are they always associated in the same individual. A certain amount of learning is unquestionably essential for a physician;—as to how much, and on what subjects, apart from that of the science itself, much contrariety of opinion exists. Dr. Stillé would undoubtedly require, as a part of his preparatory education, that a student of medicine should be well versed in the Greek and Latin languages; and yet Dr. Rush, in an introductory lecture delivered several years after that quoted by Dr. Stillé, argued that the time employed in their acquisition is mis-spent, and ought to be devoted to other objects. A gentleman, who is himself a bright example of what learning can confer upon its possessor, is content to say that "the education of the youth who is intended for the medical profession should be essentially that adapted for the well educated gentleman," which is probably as near an approximation to the point as we are likely to arrive at.

It is a misfortune that in all attempts at reform the errors proposed to be corrected and the advantages to be gained by the change, are equally exaggerated in the minds of those engaged in the enterprise. They too often see only what seems *desirable:* the *practicable* is overlooked, and they forget that alteration is not always improvement.

Whatever may be said of the system pursued in France,—in point of *learning* the members of the medical profession cannot be compared with those of Britain. Even in professional qualifications, with a few bright exceptions, they are behind our own countrymen. Too often, their views and attainments are limited to some specialty, which, in the estimation of the individual, comprises the whole circle of knowledge.

The lecture before us derives importance at the present moment, beyond what is ordinarily due to a well written essay by an estimable individual, from the spirit of reform which has gone abroad among the profession in this country, the promptings of which are to be manifested in the proceedings of the National Medical Convention to assemble in this city in May next. From the diverse objects contemplated by those who are to take a part in the proceedings of the Convention, and the discordant views entertained and already expressed on many points, it is difficult to form any idea of what will be the results of its deliberations. If all utopian schemes be discarded, and none but such as are practical adopted, good may result. For ourselves, we neither anticipate so much good, nor apprehend so much confusion and evil from its proceedings, as many of those who are arranged on opposite sides. None have laboured more earnestly than ourselves, in times past, to elevate the standard of medical education in the United States, nor does any one more sincerely desire it now. On this point our feelings have undergone no modification in the lapse of time and the change of circumstances; but our knowledge of the difficulties that lie in the way is increased, and with it our hopes and anticipations of great results are diminished.

The burden of complaint in the lecture before us, as well as in the mouths of all who are most earnest in the movements on this subject is, that the courses of instruction given in the colleges are not sufficiently numerous and complete, and that the standard of graduation is too low,—forgetting altogether that a very large number of the practitioners throughout the country attend no lectures at all, and a still larger number never graduate or submit to any examination whatever. Now if all could be induced to attend lectures and demonstrations, and undergo an examination, no one can doubt that it would be the greatest of all reforms, as regards the interests of humanity, and the honour of our profession. The great and lamentable error of the existing state of things is, not that they who graduate are insufficiently instructed, but that so many engage in practice who have received little or no instruction at all. It is a serious question, whether any measures that would subject the student to a greater expenditure of time and money to obtain a degree, would not increase the number of uneducated practitioners, and in this way reduce rather than elevate the general standard of medical education in the country. If some means can be devised to require every man to submit to examination before entering into practice, it will do more to improve the respectability and usefulness of the profession, than any and all other means we have heard suggested for that purpose.

The practical scheme of improvement in medical education proposed by Dr. Stillé, at least as a beginning, notwithstanding his somewhat enthusiastic reasoning on the subject, is far from being so unfeasible as many we hear proposed. It is as follows "I will venture to state what reforms appear to me most desirable, and, at the present time, most feasible. And first, the prolongation of the lecture term from four to six months, without greatly increasing the number of lectures beyond that already delivered, in order to give the student time to think, read and attend the hospitals. Subsequently the entire term of study might be prolonged from two to three winters; an attendance, is now required on two courses only of subjects named in the existing curriculum, and the student restricted during the first year to the lectures on anatomy, chemistry, physiology, and materia medica. In the second or third year, the instruction might include several departments not now studied, such as general pathology, morbid anatomy, medical jurisprudence and hygiene, of which one course might for the present suffice."

We have no doubt whatever that this is quite as much as, and probably more than, can be effected; but will the advocates of reform be content to stop here? Will not many of those who have recently engaged in the cause with hot haste, be like the boy with the filberts,—grasp more than is compatible with success? We greatly fear so.

*Contributions to the Natural History of the Alligator, (Crocidilus Mississippiensis) with a Microscopic Addendum.* By Bennet Dowler, M. D.

The New Orleans Medical and Surgical Journal of November, 1846 contains an article under this head, which has been reprinted and constitutes a pamphlet of thirty pages, abounding in curious and interesting matter.

We have several times had occasion to notice the labours of Dr. Dowler, and to speak of the strikingly original and pains-taking character of his researches. The present essay, although on a widely different subject from those heretofore noticed, manifests the same independent and investigating spirit, and from the statements it contains in relation to the anatomical structure and habits of the alligator, so different from the history of the animal given by naturalists, must be regarded with much attention by all who feel interested in this branch of natural science. Contrary to what has been asserted, Dr. D. maintains that the alligator is identical with the crocodile; and that the varieties found on the Nile, the Ganges, the Mississippi, &c., exhibit only the modifications caused by climate.

Speaking of the Alligator, he observes: "The *upper jaw* is wider than the under, which it overlaps. The latter has forty teeth, none of which are *grinders*, as asserted by Professor Owen —none are cutting or incisor teeth, as they are described by Goldsmith. The teeth of the upper jaw are similar in number and structure." * * * "The form and situation of the dental organs, together with the osteological configuration of the jaws, render *grinding* operations quite impossible. The animals found in the stomachs of alligators, show that their prey is killed by penetrating bayonet-like wounds, and swallowed without mastication. The crushing and prehensory power of the jaws and teeth is as remarkable as it is unquestionable.

"Herodotus, Pliny, Aristotle, and many more modern *savans*, including certain French academicians, assert that the upper jaw moves independently of the head, though both are known to constitute a continuous mass of bone, without any flexible articulation. I have for hours forced the jaws assunder by levers, elevating the upper jaw, and with it the head. The cranium, and the superior maxillary bone constitute a continuous pyramidal mass of osseous matter, the base of which is the skull, and the apex the muzzle."

The author gives a very full and graphic account of the various organs of the animal's body, in which he often differs widely from former historians; and likewise an interesting account of its habits, in which he certainly represents it in a much more *amiable* light than we have been accustomed to view it.

---

*Hand-Book of Human Anatomy, general, special, and topographical. Translated from the original German of Dr. Alfred Von Behr, and adapted to the use of the English Student.* By JOHN BIRKETT, Fellow of the Royal College of Surgeons of England, and Demonstrator of Anatomy at Guy's Hospital. Demi 8vo. pp. 487. Lindsay and Blakiston, Philadelphia, 1847.

This is a reprint of a recent London publication, and time enough has not yet elapsed since it assumed the English dress to determine the rank it is to hold among the treatises on the same subject now before the profession. As anatomists and experimental physiologists, our German brethren have no superiors at the present day. They possess, besides genius, a patient and plodding industry that accomplishes wonders in matters of detail, and consequently it is to them that we are indebted for a large amount of our present advanced knowledge of miscroscopical Anatomy. The present work, although excessively condensed, contains many evidences of the traits we have mentioned. It was not originally published as an isolated volume, but is one of a series entitled "The Pocket Encyclopædia of the Medical Sciences," by Dr. Von Behr, and Dr. Minding, and now in course of publication at Erlangen. We cannot say that it is calculated to supersede the more extended treatises on the various subjects it comprises, but as a *remembrancer* for the physician, and guide to the student while prosecuting his studies in practical anatomy, it appears to us to be exceedingly well adapted.

The American publishers have done credit to the work and to themselves, by the superior manner in which it is brought out. Too often an author is insulted and his book degraded by the beggarly dress in which it is sent forth by the publishers—a charge, we are happy to say, rarely applicable of late years to Philadelphians.

*Summary of the Transactions of the College of Physicians of Philadelphia.* Vol. 1. From Nov. 1841 to August 1846. 8 vo. pp. 461.

During the last five years it has been the custom of the College of Physicians to publish quarterly summaries of its proceedings. These have now become sufficiently extensive to make a good sized volume, and are bound up in that form for the use of the members and such others as may desire to purchase it.

Beside reports on most of the branches of the science, made at stated periods by Committees, these "transactions" contain the discussions of the members on the various interesting points presented before the College, either in the reports or oral communications, and express, generally, the views and experience of the speakers on the doctrines and practice of the day. On various occasions we have enriched our pages with extracts from these quarterly reports, and find that many of our brother journalists have done the same, which is sufficient evidence of the value of the matter they contain.

